# The effect of spatial organization of targets and distractors on the capacity to selectively memorize objects in visual short-term memory

**DOI:** 10.5709/acp-0160-x

**Published:** 2014-09-30

**Authors:** Aymen Ben Abbes, Emmanuelle Gavault, Thierry Ripoll

**Affiliations:** 1 Higher Institute of Human Sciences of Tunis, University of Tunis El Manar, Tunisia; 2 National Center for Scientific Research, Laboratory of Cognitive Psychology, Aix-Marseille University, France

**Keywords:** visual short-term memory, selective attention, spatial organization, cueing

## Abstract

We conducted a series of experiments to explore how the spatial configuration of objects influences the selection and the processing of these objects in a visual short-term memory task. We designed a new experiment in which participants had to memorize 4 targets presented among 4 distractors. Targets were cued during the presentation of distractor objects. Their locations varied according to 4 spatial configurations. From the first to the last configuration, the distance between targets’ locations was progressively increased. The results revealed a high capacity to select and memorize targets embedded among distractors even when targets were extremely distant from each other. This capacity is discussed in relation to the unitary conception of attention, models of split attention, and the competitive interaction model. Finally, we propose that the spatial dispersion of objects has different effects on attentional allocation and processing stages. Thus, when targets are extremely distant from each other, attentional allocation becomes more difficult while processing becomes easier. This finding implicates that these 2 aspects of attention need to be more clearly distinguished in future research.

## Introduction

The visual working memory system, responsible for the short-term retention and the manipulation of visual information, is subject to severe storage capacity limitations (Cowan, 2001; Jiang, Olson, & Chun, 2000; Pashler, 1988; Phillips, 1974; Rensink, 2000; Vogel, Woodman, & Luck, 2001). To overcome this constraint, our visual system uses mechanisms which enable us to select the relevant information to be stored. In this context, many studies clearly show that this selection and transfer of information in visual short-term memory (VSTM) are controlled by bottom-up and top-down attentional processes (Cowan & Morey, 2006; Henderson & Hollingworth, 1999; Schmidt, Vogel, Woodman, & Luck, 2002; Vogel, Woodman, & Luck, 2005). However, even though we know that attending to a particular location or object improves its transfer into VSTM, we do not know how attentional allocation to multiple locations leads to the transfer of multiple corresponding objects in VSTM. This study was conducted in order to explore this capacity. Indeed, we propose to test whether the VSTM capacity is reduced when there is more than one to-be-stored object and when these objects are interspersed with distractors. Furthermore, we try to explore the effect of the spatial distribution of these objects between distractors on VSTM capacity. Such questions are ecologically important since the to-be-stored objects are frequently combined with other irrelevant objects in natural environment. As a consequence, the evaluation of VSTM capacity in a noisy environment is relevant and informative. Moreover, these questions are theoretically relevant, as they involve the complex relationships which link attention to VSTM (Awh, Vogel, & Oh, 2006).

As said above, the spatial organization of objects is one of the variables we investigate since, as Jiang et al. (2000) suggest, spatial configuration is the framework supporting VSTM. Thus, any change in the spatial configuration of objects is accompanied by a facilitation or interference in selecting and storing these objects. The predictions we can make concerning the impact of such manipulation are related to a huge theoretical background. Indeed, there is no consensus about the characteristics of selective spatial attention, and there are opposite views that describe the impact of the spatial organization of objects on the capacity to select them. In this context, we can differentiate three theoretical positions that could make different predictions according to the different types of spatial configuration of objects.

First, according to the traditional “spotlight,” “zoom lens”, and “gradient” theories, an attentional “beam” facilitates the processing of only the stimuli that are located within its focus (Eriksen & St. James, 1986). Attentional facilitation is limited to a single region, excluding any possibility to prioritize targets dispersed among distractors (Heinze et al., 1994; McCormick & Klein, 1990). A clear prediction from this theoretical approach is that identifying and memorizing targets should be more difficult when dispersion increases: Global performance should be maximal when targets are contiguous and clearly separated from distractors. When targets and distractors are fully intermixed (high level of dispersion), the cueing of targets should not improve significantly the global performance.

Although a lot of empirical data are consistent with the unitary conception of attention, several recent studies, taking an approach different from spotlight theory, support the hypothesis that attention can be simultaneously applied over multiple distant locations or objects (Awh & Pashler, 2000; Bundesen, Habekost, & Kyllingsbaeck, 2005; Gobell, Tseng, & Sperling, 2005; McMains & Somers, 2005; Ripoll, Albert, Ben Abbes, & Gavault, 2008). If multiple foci of attention at different locations can be allocated simultaneously, it should be possible to take advantage of multiple location cues in a visual memory task. And yet, a clear prediction from this theoretical approach of attention remains difficult for two main reasons (for more details, see Wright & Richard, 2003). First, if we consider experimental paradigms used to study split attention, in most cases, only two attentional locations were cued (and so there is a real lack of empirical and coherent data for visual stimuli involving more than two targets). Second, it is gene-rally accepted (apart from McMains & Somers, 2005) that the division of attention has a cost. Consequently, as in the unitary spotlight approach, the hypothesis of multiple attentional foci would predict that performance decreases as dispersion increases. Given the difficulty to estimate quantitatively how the performance should be affected by the splitting of attention, no clear empirical and crucial prediction can differentiate the unitary from the multi-focal conception of attention.

A third interesting approach, the competitive interaction model (Bahcall & Kowler, 1999; Caputo & Guerra, 1998; Cutzu & Tsotsos, 2003; McCarley, Mounts, & Kramer, 2004; Mounts & Gavett, 2004), inspired by the biased competition model of Desimone and Duncan (1995), leads to different predictions. According to this model, objects compete with one another for representation within the visual system. Consequently, attentional selection allows to enhance processing of the selected objects at the representational expense of other objects in the near visual environment. The competition between two objects is maximal when these objects are in close spatial proximity and decreases when they are distant because these objects compete only to the extent that they draw from the same pool of receptive fields. For example, Bahcall and Kowler (1999) showed that the comparison of two targets is both more rapid and accurate when the distance between them increases and when the targets are separated by one or more distractors. Recently, Franconeri, Alvarez, and Enns (2007) observed the same effect, that is, accuracy in a visual search task diminishes as the spatial separation between targets decreases.

To summarize, in the following five experiments, we tried to evaluate the VSTM capacity using a visual environment in which targets and distractors were simultaneously present. We investigated how the targets/distractors organization determined both the deployment of attention and the capacity to select and memorize the targets.

In our paradigm, the participants were presented with a circular array of eight objects: four distractors and four targets. Their task was to memorize the identities and the locations of the targets while ignoringthe distractors. Time of exposure was short (150 ms), limiting the possibility to move the eyes among the elements of the circular array. Furthermore, targets and distractors were physically similar: No feature properties (such as color or abrupt onsets) allowed for pre-attentively distinguishing targets from distractors. Only spatial information, given at the beginning of each trial, could be used to prioritize targets. Thus, the capacity to select targets among distractors efficiently will only depend on the characteristics of attentional deployment.

## Experiment 1

In this first experiment, we investigated how temporal factors and stimulus organization (i.e., the dispersion level of the targets) determined performance in a VSTM task. Participants had to memorize four targets presented among four distractors. Targets differed from distractors only in their location and their history. The four distractors were presented prior to the presentation of targets. Thus, these distractors played the role of cues by informing the participants about the future locations of the targets: Targets appeared where distractors were absent. After a variable delay during which neither distractors nor targets were present, both objects appeared simultaneously for 150 ms.

Three main factors were manipulated. The first two factors concerned temporal aspects. First, between subjects, we varied the distractors presentation time (100, 300, or 500 ms). These values have been chosen because they concord with the classical estimation of the time necessary for an endogenous deployment of attention (Müller & Rabbitt, 1989). Such time manipulation from 100 to 500 ms seems to be suited to evaluate the dynamics of the attentional process involved in this task.

The second temporal aspect we manipulated involved the inter-stimulus interval (ISI) that we varied between subjects: The time between the offset of distractors and the onset of the entire array in which the four targets and the four distractors appeared. ISI could be of 50 or 900 ms.

The last and the most crucial factor concerned the spatial organization of the targets among distractors. Four conditions of spatial organization were manipulated, as a within-subjects factor, defining four increasing levels of dispersion. In the first condition (C1), no distractor was present between the four targets. Consequently, the four targets could be considered as present within one and the same contiguous spatial area. In this case, targets’ dispersion was minimal. In the second condition (C2), targets were distributed across two non-contiguous areas, separated by at least one distractor. In the third condition (C3), targets were distributed across three non-contiguous areas; and in the last condition (C4), all targets were separated from another by one interleaving distractor. Thus, the level of dispersion increased from C1 to C4.

Finally, two control groups were distinguished. In the first group (control group 4), only the four targets were present whereas in the second group (control group 8), targets and distractors were both present, and participants did not have information about targets locations. As a consequence, they could not distinguish targets from distractors.

### Method

#### Participants

A total of 80 undergraduate students (43 male and 37 female; *M*_age_ = 24.6, range 19-27) volunteered for this experiment, 10 in each group (“control group 4”, “control group 8”, and “preview groups”: 100/50 [100 ms corresponding to the distractors presentation time and 50 ms corresponding to the ISI], 300/50, 500/50, 100/900, 300/900, and 500/900). All the participants reported normal or corrected-to-normal visual acuity.

#### Apparatus

The experiment was conducted on a Macintosh computer with a 14” screen and was programmed with PsyScope software® (Cohen, MacWhinney, Flatt, & Provost, 1993). Participants were tested individually in a dimly lit room. They sat at a distance of about 70 cm from the computer screen.

#### Stimuli

**Figure 1.**
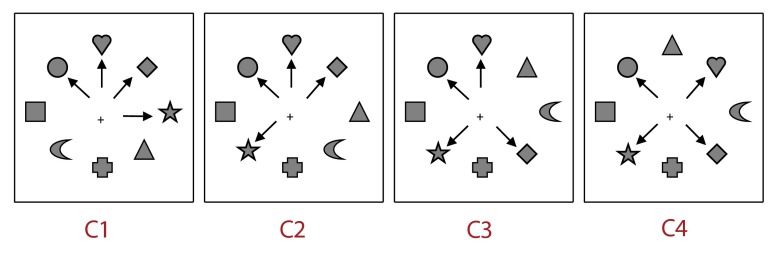
Examples of the four types of configuration used in Experiment 1: Configuration 1 (C1) in which targets were presented within a single spatial area, Configuration 2 (C2) in which targets were distributed across two spatially noncontiguous areas, Configuration 3 (C3) in which targets were distributed across three spatially noncontiguous areas, and Configuration 4 (C4) in which targets were distri- buted across four spatially noncontiguous areas. Note that arrows did not appear on the screen and just indicate the targets.

All stimuli were presented in grey on a white background. Memory arrays consisted of eight 1 × 1 cm objects (square, circle, triangle, heart, star, cross, diamond, crescent) evenly spaced on an imaginary circle with a radius of 4.7° that was centred on a fixation cross. The stimuli were arranged in a circular array display with a fixation point in its centre to ensure that retinal resolution was constant for any possible stimulus location. The spatial organization of the targets among distractors was systematically controlled. As indicated previously,four conditions of spatial organization (C1, C2, C3, and C4) were distinguished according to the locations of targets among distractors (Figure 1).

Eighty entire arrays (targets plus distractors) were built, 20 for each configuration. During the experiment, each array was presented two times as a function of a to-be-recognized object (probe) that appeared at target locations: once with an identical probe and once with a different probe.

#### Procedure

**Figure 2.**
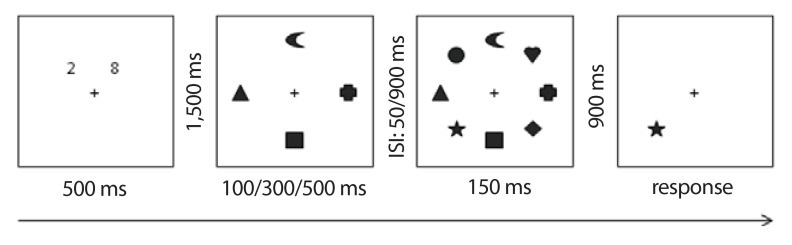
Example of a valid trial in the experimental condition (Experiment 1). Note that the numbers are used for the parallel articulatory suppression which had to be performed throughout stimulus presentation. These numbers were always presented at the same locations. ISI = inter-stimulus interval.

Each subject completed 24 practice trials and 160 experimental trials randomly presented (see Figure 2). Participants initiated each trial by pressing the space bar. They were asked to focus on the central cross for the entire trial. Each trial began with an articulatory suppression task: Two-digit numbers were presented for 500 ms at fixation, and the participants were asked to repeat them at a rate of 3-4 digits per second for the entire trial (Schmidt et al., 2002). This was followed by a 1,500-ms delay. From that moment, the procedure was quite different for the four preview groups and for the two control groups (control group 4 and control group 8).

In preview groups, the distractor objects appeared for 100 ms (100 ms condition), 300 ms (300 ms condition), or for 500 ms (500 ms condition). Following the offset of the distractors, an ISI of 50 ms or 900 ms occurred. At the end of the ISI period, the memory array consisting of the eight objects appeared for 150 ms. Participants were asked to memorize only the new objects (targets) in the memory array.

In control group 8, the full memory array (four targets and four distractors) appeared for 150 ms. In control group 4, only the four targets were presented.

The offset of the memory array was followed by a delay of 900 ms, in order to ensure testing the VSTM and not the sensory memory. The probe object then appeared and was always at target locations. The participants responded on a standard keyboard by pressing the “q” button to indicate that the probe object was identical to the target at the same location and the “p” button if probe and target were different. The probe and target shape were identical in half of the trials.

### Results

The percentage of correct responses was calculated for each condition. Mean accuracies are reported in Table 1.

**Table 1.** Percentage of Correct Responses as a Function of Configuration Type and Conditions

ISI	Times	Configurations				
		1	2	3	4	Means
50 ms	100 ms	69 ± 02	69 ± 02	63 ± 04	68 ± 04	67 ± 03
	300 ms	69 ± 03	70 ± 04	70 ± 03	78 ± 02	71 ± 03
	500 ms	76 ± 03	68 ± 02	69 ± 02	80 ± 01	73 ± 02
Means		71 ± 03	69 ± 03	67 ± 03	74 ± 03
900 ms	100 ms	73 ± 03	70 ± 03	68 ± 02	79 ± 02	73 ± 02
	300 ms	79 ± 02	73 ± 02	69 ± 02	79 ± 02	75 ± 02
	500 ms	79 ± 01	71 ± 02	75 ± 03	80 ± 02	76 ± 02
Means		77 ± 02	71 ± 02	71 ± 02	79 ± 02	
Control 4		75 ± 02	81 ± 02	76 ± 03	80 ± 02	78 ± 02
Control 8		65 ± 02	61 ± 03	65 ± 02	64 ± 02	64 ± 02

*Note* Data are shown ± standard errors. ISI = inter-stimulus interval.

First, data were submitted to an analysis of variance (ANOVA) with Type of Presentation as between-subjects factor (comparison between control group 4, control group 8, and the preview group) and with Type of Configuration (C1, C2, C3, and C4) as within-subject factor. This analysis revealed a significant main effect of the type of presentation, *F*(2, 77) = 12.89, *p* < .001, η_p_^2^ = .25. In order to analyze this global effect, planned comparisons were conducted. Performance of control group 4 was better than performance of control group 8, *F*(1, 77) = 24.80, *p* < .001, η_p_^2^ = .24. More interestingly, participants of preview groups who previewed the distractors had better performance than participants of control group 8, *F*(1, 77) = 16.00, *p* < .001, η_p_^2^ = .17. Nevertheless, the performance of participants of the preview groups did not reach the level of performance of participants of the group control 4, *F*(1, 77) = 6.30, *p* = .01, η_p_^2^ = .07. Second, the effect of the type of configuration was significant, *F*(3, 231) = 3.10, *p* = .02, η_p_^2^ = .04. This effect was modulated by the type of presentation, *F*(6, 231) = 2.40, *p* = .02, for the effect of interaction between the Type of Configuration and the Type of Presentation.

Subsequently, in order to analyze the effect of spatial configuration, we conducted an ANOVA, with Type of Configuration (C1, C2, C3, and C4) as within-subject factor, separately for each group.

An ANOVA with the Type of Configuration as the only within-subject factor applied to each control group revealed no significant effect of the type of configuration in control group 4 (*p* = .50) as well as in control group 8 (*p* = .45).

In preview groups, an analysis with the Type of Configuration as within-subject factor, and ISI and Distractors Presentation Time as between-subjects factors was conducted.

The main effects of distractors presentation time and ISI were significant; *F*(2, 54) = 3.40, *p* < .05, η_p_^2^ = .11; and *F*(1, 54) = 5.90, *p* < .05,η_p_^2^ = .09, respectively. The interaction between these two factors was not significant (*p* = .84). Performance was better with a 900-ms ISI than with a 50-ms ISI. Furthermore, planned comparisons revealed that participants who previewed the distractors for 500 ms performed better than participants who previewed the distractors for 100 ms, *F*(1, 54) = 6.48, *p* < .01, η_p_^2^ = .10; but neither the difference between the 500 ms preview group and the 300 ms preview group nor the difference between the 300 ms preview group and the 100 ms preview group were significant; *F*(1, 54) = 0.60, *p* = .43; and *F*(1, 54) = 3.00, *p* = .08, respectively.

**Figure 3.**
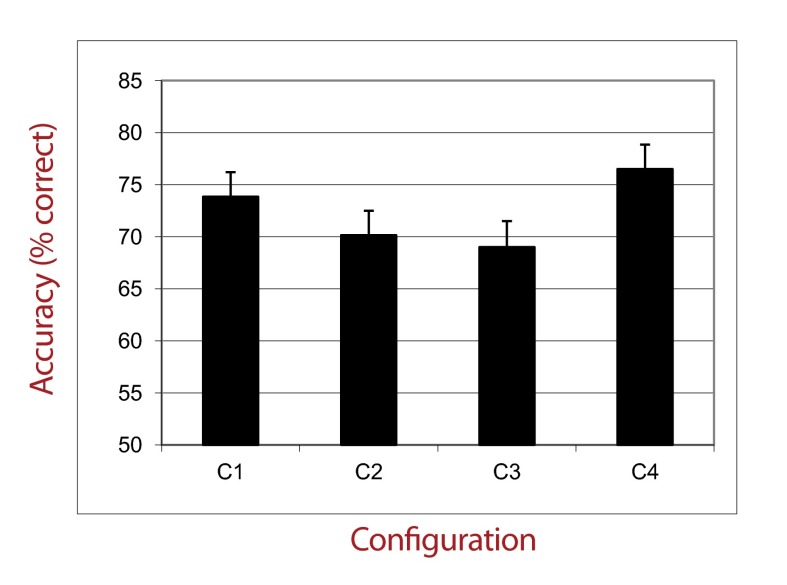
Percentage of correct responses in experimental groups as a function of Configuration Type (Experiment 1).

This ANOVA also revealed a strong main effect of the type of configuration (see Figure 3), *F*(3, 162) = 22.10, *p* < .001, η_p_^2^ = .29. We conducted a post hoc analysis (Fisher’s LSD) which showed that participants achieved better performances in condition C4 than in conditions C1, C2, and C3 (all *ps* < .001). Furthermore, performance in condition C1 was better than performance in conditions C2 and C3 (*p* < .001). No difference was found between performance in conditions C2 and C3 (*p* = .37). The interaction effect between Type of Configuration and Distractors Presentation Time (*p* = .09) as well as between Type of Configuration and ISI (*p* = .63) were not significant.

### Discussion

Results clearly show that participants can take advantage of the prior presentation of distractors to selectively process and memorize four targets among distractors. This selective processing is quite remarkable since, if participants are given sufficient time to accurately encode four targets locations (e.g., 500 ms in one of the preview conditions), performance is close to that in control group 4. For configurations C1 and C4, the performance of preview groups was as good as performance in control group 4, as if participants could perfectly ignore the distractors.

The selective processing of targets is strongly modulated by temporal parameters. After a period as short as 100 ms, participants began to take advantage of the preview. This benefit is maximal for a presentation time of distractors of 300 ms and 500 ms. By increasing distractors presentation duration, participants can extract more information about future target locations and can allocate their attention to these locations more accurately.

The ISI manipulation shows that attentional deployment onto target locations cannot result from an automatic capture by target onset. Indeed, an abrupt onset by the targets might have automatically captured attention with a 50-ms ISI but not with a 900-ms ISI (Theeuwes, 1991; Yantis & Hillstrom, 1994). Yet, the results showed a small but significant difference in favor of a 900-ms ISI: Endogenous attention is, thus, clearly involved in this capacity to memorize targets among distractors. This finding is of some importance because it shows a clear difference with a similar phenomenon that Watson and Humphreys (1997) have revealed in visual marking experiments. We need to remember that in visual marking, a preview benefit is observed only when new items onset. Indeed, the preview benefit was abolished when the new items were isoluminant with the background (Donk & Theeuwes, 2001) or when old items disappeared for more than 400 msbefore appearing again with new items. In our paradigm, targets and distractors onset simultaneously (as is the case in natural environments) preventing any sort of sensory facilitation. Thus, these findings support previous results highlighting the existence of top-down goal-based mechanisms that bias inputs into VSTM (Gavault & Ripoll, 2004; Schmidt et al., 2002). Our findings, thus, complement earlier studies showing that bottom-up factors (peripheral cues, popout, perceptual organization) have an impact on memory storage (e.g., Woodman, Vecera, & Luck, 2003).

#### Spatial organization effect

The spatial organization effect takes an unexpected and very interesting form. First, there is not a linear relation between accuracy and target dispersion: Accuracy is higher for the minimal and maximal levels of dispersion (conditions C1 and C4) and lower for intermediate levels (conditions C2 and C3). Second, performance is the best when dispersion is maximal. In this case, the performance reaches a very high degree of accuracy in a way that the performance in condition C4 does not differ from performance in control group 4. This last result is especially important because it shows that the presence of distractors between each target does not present a real difficulty for the visual system.

We carried out an additional analysis to test whether the accuracy varied with the relative spatial position of the probed target. We did not observe any effect of such factor. In the same line, we did not find any dispersion effect in the two control groups. We could then deduce that the dispersion effect in experimental groups is not a consequence of variation in the ability to identify and memorize targets at different locations along the circular array. Moreover, since the location of targets is exactly the same in preview groups and control group 4, we can conclude that attentional parameters are responsible for the observed dispersion effects. Similarly, Cutzu and Tsotsos (2003), in a quite different visual matching task, found that effects of inter-target separation disappeared when attention was not cued before the onset of the circular array. From a theoretical point of view, the absence of a dispersion effect in control group 4 is particularly interesting because it suggests that this effect is not a consequence of any low-level sensory masking effects (lateral masking or crowding), but is contingent upon the spatial distribution of attention within the display. In other words, it is not the intrinsic property of targets’ organization which is responsible for the dispersion effect but the kind of attentional distribution that the targets/distractors organization involves.

On the whole, this general pattern of results does not concord with the unitary conception of attention since the performance is the highest when dispersion is maximal. Such results seem relatively compatible with the competitive interaction model. It explains perfectly that the highest level of performance is observed in condition C4. In this condition, targets are not in close spatial proximity and they do not draw on the same pool of receptive fields. As a consequence, their competition and so, their mutual interference were reduced. In this condition, encoding and consolidation in VSTM are optimal because the distance between targets was maximal. Nevertheless, an aspect of these data is not consistent with the competitive interaction model. Indeed, the performance is higher in condition C1 than in conditions C2 and C3 whereas the distance between targets is minimal in condition C1. We will discuss this discrepancy later.

A full understanding of this pattern of findings will require some methodological considerations. One of the potentially most important methodological concerns is linked to the circular organization of the eight objects in the final array. Only two variants of condition C4 can be created (diamond organization and square organization) whereas many more different variants are possible for conditions C1, C2, and C3. Consequently, the frequency of the two variants of condition C4 is higher than that of the several possible variants of conditions C1, C2, and C3 in such a way that a simple frequency of spatial pattern effect could explain the surprisingly high level of performance in condition C4. Therefore, we designed an experiment for neutralizing this potential bias by using only two variants for each type of configuration (C1, C2, C3, and C4).

## Experiment 2

The aim of Experiment 2 was to make sure that the configuration effects found in Experiment 1 were not due to a possible frequency effect.

### Method

#### Participants

Twenty four undergraduate students (10 male and 14 female; *M*_age_ = 22.9, range 20-26) with normal or corrected-to-normal vision participated in this experiment which concerned only one preview condition (300/900).

#### Apparatus

The same apparatus as in Experiment 1 was used.

#### Stimuli

We chose only two versions for each type of the four conditions of configuration.

#### Procedure

The procedure was the same as in Experiment 1 but we restricted the comparison to the preview group. We selected an ISI of 900 ms and a distractors presentation time of 300 ms because the configuration effects were very clear with these temporal parameters.

### Results

**Figure 4.**
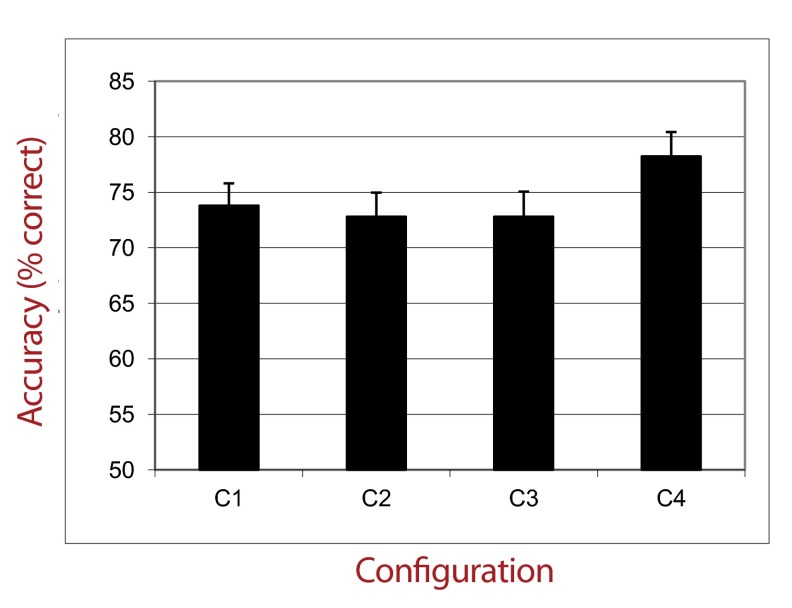
Percentage of correct responses in experimental groups as a function of Configuration Type (Experiment 2).

The percentage of correct responses was calculated for each type of configuration. Mean accuracies are plotted in Figure 4. A repeated measures ANOVA with Type of Configuration as within-subject factor showed a significant main effect, *F*(3, 69) = 3.56, *p* < .05, η_p_^2^ = .13. Post hoc tests (Fisher’s LSD) yielded significant differences between performances for Configuration 4 and those for the other configurations (Configurations 1, 2, and 3; *ps* < .01). No difference was detected when contrasting C1 with C2 and C1 with C3 (*p* = .397). Finally, performances with C2 did not differ from performances with C3 (*p* = .95).

### Discussion

Taken as a whole, the pattern of results is very similar to the pattern of results found in the previous experiment. Performance is better in condition C4 than in any other condition. Consequently, a possible frequency bias cannot explain the high level of performance observed in condition C4.

Nevertheless, there is still another important factor, confounded with the level of dispersion, which could explain the high level of performance in condition C4: The level of dispersion of this condition is maximal but its organization has a special and unique characteristic. Indeed, the two configurations in condition C4 have the status of a good form because the four targets are organized either in a square-object shape or in diamond-object shape with perfect symmetry. Thus, level of dispersion and form goodness are confounded factors. Such a confounding is problematic since previous research has shown that perceptual organization in general can bias the storage of visual information (Woodman et al., 2003) or the capacity to track moving objects (Yantis, 1992). So, performance in condition C4 could be very high not because the level of dispersion is maximal, but because the good form of targets organization allows the subjects to allocate their attention more easily and more accurately to target locations. The aim of the next experiment was to dissociate the impact of the “good form” from that of dispersion of targets. To do so, we contrasted a condition in which targets are organized according to a regular spatial configuration to another one in which this spatial organization is considered as perceptually irregular. Since condition C4 allows only regular configurations, we increased the number of distractors to overcome this problem. This manipulation allows at the same time to design two types of spatial configurations which could or could not have the good form’s property and at the same time maintain the same high level of dispersion (i.e., at least one distractor between each target). In such a way, we will be able to dissociate these two factors and to evaluate them separately.

## Experiment 3

### Method

#### Participants

Twelve undergraduate students (seven male and five female; *M*_age_ = 24.3, range 21-26) with normal or corrected-to-normal vision volunteered for this experiment.

#### Apparatus

The same apparatus as in Experiment 1 was used.

#### Stimuli

**Figure 5.**
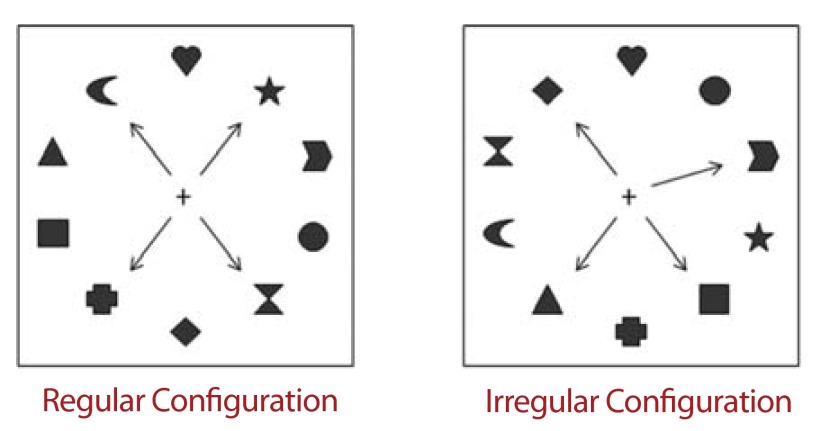
Example of regular and irregular C4 configurations used in Experiment 3. Note that arrows did not appear on the screen and are just used here to indicate the targets.

The only difference to the previous experiments is that two additional objects were used: The memory array contained six distractors and four targets. Targets were separated by one or two distractors. Only condition C4 was manipulated and could take two different forms: a regular configuration (good form of targets: square and diamond configurations) or an irregular configuration (Figure 5). A total of 80 entire arrays (targets plus distractors) were built, 40 for each configuration.

#### Procedure

The procedure was exactly the same as in preview groups of Experiment 1. As in Experiment 2, ISI was of 900 ms, and the distractors presentation time was of 300 ms.

### Results

Mean accuracies were calculated as a function of the type of configuration. A repeated measures ANOVA with Type of Configuration as within-subject factor yielded a main effect of this factor, *F*(1, 11) = 12.60,*p* < .01, η_p_^2^ = .53. Performance was better when targets were presented in a regular configuration than when they were presented in an irregular configuration.

### Discussion

The best performance in the “good form” condition shows that it is easier to select and memorize targets among distractors when these targets can be organized in a good form. Such a finding is consistent with the “chunking” account suggesting that the VSTM capacity can be improved by grouping items into an integrated object or shape. This combination leads to a better selection and then, to a more sophisticated and complete VSTM storage (Jiang et al., 2000; Miller, 1956).

Given that attention plays a crucial role in the selection of visual information in VSTM, one can infer that it is easier to allocate attention to different locations when these locations are organized in a good form. However, the high performance observed in condition C4 in the two previous experiments cannot be exclusively explained by this factor. Indeed, performance in the irregular configuration condition remains relatively high (77%). Such a result is all the more surprising as the spatial distance between locations is smaller than in the previous experiments. Consequently, even if Gestalt principles of organization play a role in the capacity to select targets among distractors (e.g., Yantis, 1992), the dispersion level of the targets seems to be a crucial determinant of performance. Globally, these results suggest that memorizing targets is easier when they are distant and separated from each other by distractors whenever these objects are or are not organized in a good form.

## Experiment 4

The high performance in condition C4 seems very reliable, and the best way to explain this superiority is most probably linked to the fact that the level of dispersion is maximal in this condition. As the competitive interaction model assumes, the competition between attended objects is inversely related to their spatial distance. In condition C4, this spatial distance is maximal because targets are separated from each other by a distractor. As a consequence, competition is minimal and VSTM storage is facilitated because the encoding of each target does not interfere with that of the other targets. Nevertheless, this theoretical model cannot explain the high performance in condition C1.

The first explanation of this discrepancy is methodological. We cannot exclude that an ocular saccade contributed to the performance before the onset of the memory array in condition C1. An ocular movement could facilitate the task only in this condition. One of the aims of this experiment is to evaluate the possible effect of ocular movements.

The second explanation of this general pattern involves a more theoretical analysis. Many authors suggested that the concept of attention may involve distinct aspects (Huang & Pashler, 2007; Vogel et al., 2005). Probably the most obvious distinction relates to two different aspects of attention which are frequently confounded: selection and processing. When Desimone and Duncan (1995) laid down the basis of the biased competition model, they described two basic phenomena in relation with the nature of visual attention. The first basic phenomenon was the ability to filter out unwanted information or/and to select relevant information. The second was the limited capacity for processing information. In general, selectivity is conceived as a way to overcome the limited capacity of the visual system (Broadbent, 1958). Thus, attention is involved in the selection of relevant objects, this selection being a necessary condition for optimizing the processing of these objects. In our task, performance depends on both the difficulty to orient attention to target locations (selection) and the difficulty to consolidate their visual traces in VSTM (processing). In concrete terms, the participants have first to allocate their attention to the target locations and, once attention has been allocated towards them, every sensory trace of target has to be consolidated to reach a stable state in VSTM. Consequently, the difficulty to allocate attention to target locations and the difficulty to consolidate their visual trace in VSTM could vary differently from condition C1 to condition C4. There is no reason to assume that the spatial dispersion determines the difficulty to allocate attention to target locations and the difficulty to encode and memorize them in the same way. As many previous studies showed (e.g., Heinze et al., 1994; McCormick & Klein, 1990; Posner, Snyder, & Davidson, 1980), it is easier to allocate attention to one contiguous spatial area (as in condition C1) than to allocate attention to several spatially non-contiguous areas (conditions C2 to C4). Conversely, the consolidation process could be more difficult when targets are spatially close because each target can compete with the others for its representation in VSTM as assumed by the competitive interaction model. So, the global pattern we observed could result from the combination of these two different effects of target/distractor spatial organization: the effect on selection and the effect on consolidation.

We decided to introduce a strong visual contrast (targets were black and distractors were red) that would allow a very easy distinction between targets and distractors. Such a manipulation cannot perfectly neutralize the difficulty to allocate attention in the different conditions of dispersion but it should reduce this selection difficulty considerably. Having neutralized the influence of attention, we should theoretically only observe a positive linear relation between dispersion and performance if the targets are better consolidated as the spatial distance between them increases.

In the same line, we had already seen in Experiment 3 that the good form allowed to improve the attentional capacity. If we obtain the positive linear increase of performance mentioned above, we could not explain such result only by the good form factor. Indeed, this factor could not explain the superiority of the performance in conditions C2 and C3 (in which no good form effect was suspected) compared to condition C1. Such finding would be due exclusively to the increase of the spatial distance between targets.

### Method

#### Participants

Twenty-four undergraduate students (seven male and 17 female; *M*_age_ = 21.7, range 19-23) with normal or corrected-to-normal vision volunteered for this experiment, eight in each group (control group 8, and 100, and 500 ms preview groups).

#### Apparatus

The same apparatus as in Experiment 1 was used.

#### Stimuli

The stimuli were the same as in Experiment 2, unless otherwise noted. Targets differed from distractors by their colors: Targets were black and distractors were red.

#### Procedure

The procedure was the same as in Experiment 1. We only tested control group 8 and two preview groups distinguished by the presentation time of distractors (100 or 500 ms). The ISI was constant (900 ms).

### Results

**Figure 6.**
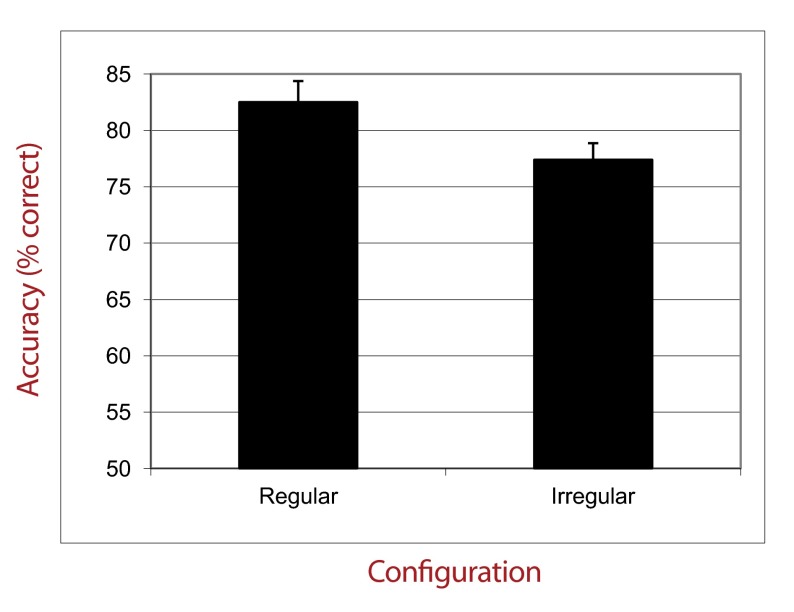
Percentage of correct responses as a function of Configuration Regularity.

The percentage of correct responses was calculated for each group. Mean accuracies are presented in Figure 6.

First, data were entered in an ANOVA with Distractors Presentation Time as between-subjects factor (0 ms for control group 8, and 100 ms and 500 ms for preview groups) and Type of Configuration as within-subject factor. As in Experiment 1, the main effect of presentation time was significant, *F*(2, 19) = 10.80, *p* < .0001, η_p_^2^ = .53.

This analysis showed also a strong main effect of the type of configuration, *F*(3, 63) = 55.00, *p* < .0001, η_p_^2^ = .72. Planned comparisons revealed that participants performed better: (a) in condition C4 than in conditions C1, *F*(1, 21) = 115.00, *p* < .0001, η_p_^2^ = .84; C2, *F*(1, 21) = 44.60, *p* < .0001, η_p_^2^ = .67; and C3, *F*(1, 21) = 67.00, *p* < .0001, η_p_^2^ = .76; (b) in condition C3 than in conditions C1, *F*(1, 21) = 67.00, *p* < .0001, η_p_^2^ = .10; and C2, *F*(1, 21) = 65.00, *p* = .02, η_p_^2^ = .75; and (c) in condition C2 than in condition C1, *F*(1, 21) = 3.90, *p* < .001, η_p_^2^ = .15. The interaction between the Distractors Presentation Time and the Type of Configuration was not significant (*p* = .91).

In order to evaluate the global impact of the introduction of different colors, we compared these results with those obtained in the absence of distinctive colors (equivalent conditions of Experiment 2). This analysis showed a significant main effect of color, *F*(1, 32) = 20.40, *p* < .0001; performances being higher when color distinguishes targets from distractors. The interaction effect between Color and Type of Configuration was significant, *F*(3, 96)= 15.00, *p* < .0001. With color, performances increase in all conditions except in condition C1.

### Discussion

The presence of color has a global strong positive impact on performance. This result is consistent with previous findings (Cave & Bichot, 1999), showing that the selection of noncontiguous locations is facilitated when targets can be discriminated from distractors by a basic visual property. This result is also consistent with the data showing that target saliency can reduce classical effects of crowding and lateral-masking (Felisberti, Solomon, & Morgan, 2005). Importantly, when attention can only be feature-driven (control group 8), we found again the dispersion effects observed in the previous experiments: The performance is very good when the level of dispersion is high. Thus, the dispersion effects are not specific to a kind of attentional deployment. Furthermore, the dispersion effect appeared very early when attention was feature-driven (when distractors presentation time is only 100 ms), whereas the dispersion effect appeared later when attention was spatially and endogenously driven (see previous experiments).

Nevertheless, whatever the impact of color was, the endogenous control of spatial attention continues to play an important role since performance in preview groups is still better than performance in control group 8. Thus, it is clear that the visual system can take advantage of both spatial information and object feature information. Furthermore, the global pattern of performance associated with the dispersion effect is identical (same pattern in control group 8 and in preview groups) for spatial and object-based ways of controlling attention: The dispersion effect is a very robust and stable effect. From a methodological point of view, such finding is interesting because it shows that ocular movements before the onset of the circular array play a secondary role (if they play any role at all) and that they cannot explain the dispersion effect in our study: Ocular movements before the onset of the final array were possible in preview conditions but not in control group 8, and, yet, the pattern of results was the same in both of these conditions.

Another important finding of this experiment is that improvement of performance caused by the introduction of color is observed only in conditions C2, C3, and C4, but not in condition C1. Actually, in condition C1, the introduction of color did not improve the performance either in preview groups or in control group 8, as if participants were not able to take advantage of the color whereas they took advantage of this information in conditions C2, C3, and C4. This pattern of results is coherent with the hypothesis that color facilitated attentional deployment but had no real impact on the consolidation process. As allocating attention to one group of targets in condition C1 is already an easy task, the introduction of color did not further improve the performance in this condition. Conversely, when targets are in non-contiguous locations, the deployment of attention is more difficult and the introduction of color helped the participants to allocate attention to these locations more accurately and efficiently. As the introduction of color reduces and neutralizes the difficulty to allocate attention to target locations, the consolidation process becomes the main source of variation and, as predicted by the competitive interaction model, the consolidation is all the more difficult as the objects to-be-memorized are close to one another. In the absence of specific difficulties to orient attention to cued locations, the performance increases linearly with an increasing level of spatial dispersion.

As coherent as this interpretation might be, we did not anticipate the following result: Performance of control group 8 in condition C1 was particularly low. Such a result is quite surprising and difficult to explain since in this experiment, participants could take advantage of the color to distinguish targets from distractors. We can only conjecture that the common and contrasting color of targets leads to a fast and strong grouping in a way that the perception of the whole prevails on the separate perception of each target. This interpretation is coherent with two related phenomena. First, lateral-masking is known to increase with target-flanker similarity (Kooi, Toet, Tripathy, & Levi, 1994; Polat & Sagi, 1993) and, in this context, each target can be conceived as a competing flanker. Second, the grouping of objects increases their mutual interference (Livne & Sagi, 2007), and the similarity of color contributes to the grouping of targets. More generally, it is possible that the similarity of targets has increased their mutual competition and prevented the participants from identifying and memorizing them as independent objects.

Finally, the last result that deserves attention is the remarkably high level of performance (89%) observed in condition C4 in the preview group. This result is quite surprising because, in theory, the presence of distractors between targets should have led to a clear drop in performance. Nevertheless, the preview group and control group 4 are not really comparable since in the first case, participants know where the targets are going to appear whereas they do not have this information in the second case. Thus, control group 4 was not an appropriate reference group to evaluate the capacity of participants to ignore the distractors. The last experiment is designed to overcome this problem. To reach this objective, a very simple and direct solution consisted of cueing targets’ locations in control group 4. In this way, control group 4 and preview groups will be distinguished only by the presence of distractors.

Moreover, this new experiment served another interest. We showed in Experiment 1 that the dispersion effect did not appear in control group 4 as if the distance between targets would not be a sufficient precondition for the dispersion effect. The absence of the dispersion effect could result either from the absence of distractors or from the fact that attention was deployed in a diffuse mode when the participants do not have any spatial information about targets location (as in control group 4). Given that these two characteristics are confounded in the previous experiments, we cannot dissociate the effects linked to the way participants allocate attention before targets onset and the effects associated to the presence of distractors. If the dispersion effect is obtained when target locations are cued, we can therefore underscore the attentional nature of this effect.

## Experiment 5

### Method

#### Participants

Fifteen undergraduate students (nine male and 16 female; *M*_age_ = 22.5, range 21-26) with normal or corrected-to-normal vision volunteered for this experiment.

#### Apparatus

The same apparatus as in Experiment 1 was used.

#### Stimuli

The stimuli were the same as the stimuli used in control group 4 of Experiment 1. Cues were constructed as follows: They consisted of four 0.3 × 0.3 cm asterisks located at the future locations of the targets on the imaginary circle centered on the fixation cross.

#### Procedure

The procedure was the same as in control group 4 of Experiment 1apart from that four asterisk cues were presented during 300 ms and followed by a blank delay of 900 ms before the presentation of targets.

### Results

**Figure 7.**
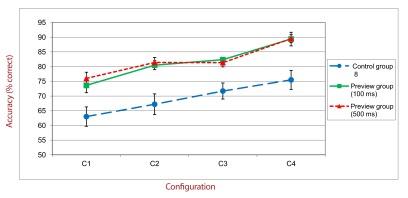
Percentage of correct responses as a function of Configuration Type and Groups (Experiment 4).

Mean accuracies were calculated as a function of the type of configuration (Figure 7). A repeated measures ANOVA with Type of Configuration as within-subject factor showed a main effect, *F*(3, 42) = 3.69, *p* < .05, η_p_^2^ = .20. Planned comparisons indicated that participants performed better with configuration C4 than with configurations C1, C2, and C3; *F*(1, 14) = 7.80, *p* < .05, η_p_^2^ = .35; *F*(1, 14) = 5.60, *p* < .05, η_p_^2^ = .28; and *F*(1, 14) = 7.10, *p* < .05, η_p_^2^ = .33; respectively. No significant difference was found between C1 and C2 (*p* = .65) as well as between C1 and C3 (*p* = .30) and between C2 and C3 (*p* = .67).

### Discussion

The cueing of target locations strongly improved performance. Even in the absence of distractors, performance was much better when participants could allocate attention only to target locations rather than, in a distributed way, to the entire circular array. This result could be easily anticipated in condition C1 because attention has to be allocated to a unique spatial area which includes the four targets. In this case, the attentional window, limited to the group of four targets, would have had less than half the size of the total area of the circular array. In this way, attentional resources would have been more concentrated in a relative small area. However, cue facilitation is more interesting in the three other conditions and especially in condition C4 in which the targets occupied the entire circular array and were fully dispersed. The high level of performance in condition C4 suggests that participants can accurately allocate attention to the four different cued locations and ignore the blank locations.

The above finding shows that, when several targets have to be processed, the endogenous deployment of attention facilitates the processing of targets even in the absence of distractors. We can explain this result in two non exclusive ways. First, it could be that the attentional window is reduced when targets have been cued because the attentional system excludes blank locations. In this case, the higher level of performance results from the reduction of the size of the attentional window. Second, the role of attention would be to reduce the mutual interference between targets. Such reduction would be optimal when targets are distant, explaining why the cueing of target locations improves performance more if the distance between targets is large. Indeed, the most interesting result is that we reproduced the same dispersion effect as in the previous experiments. The performance tends to improve as the dispersion between targets increases. This result is all the more interesting as it contrasts with the total absence of the dispersion effect observed when no cue guides attention deployment as in control group 4 of Experiment 1. Such a finding is yet another clear demonstration that the dispersion effect is not a consequence of a low-level sensory masking, but is contingent on the spatial distribution of attention within a display.

**Figure 8.**
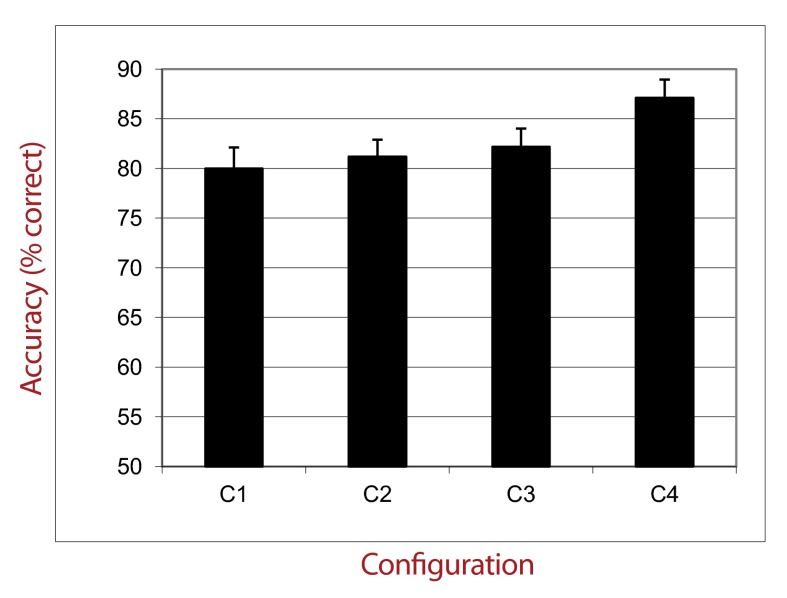
Percentage of correct responses as a function of Configuration Type (Experiment 5).

## General discussion

Generally, the results we obtained revealed a very good capacity to select and memorize four targets among distractors. This is consistent with the estimation of VSTM capacity (around four objects; Vogel et al., 2001) and the number of different objects or locations that could be attended during one ocular fixation (Franconeri et al., 2007) or tracked in visual tracking experiments (Pylyshyn, 1989; Shim, Alvarez, & Jiang, 2008). This link between attention and VSTM has been confirmed by fMRI results showing strong cerebral convergences between memory and attentional process (Cavanagh & Alvarez, 2005; Todd & Marois, 2004). In line with some VSTM studies (e.g., Sperling, 1960), these results show that the visual system can process and memorize four objects in VSTM, whatever their location in space and without being strongly impaired by the presence of distractors. Moreover, they are consistent with the findings of previous studies suggesting that the targets’ perceptually “good” spatial organization allows better selection and storage in VSTM (Jiang et al., 2000; Vidal, Gauchou, Tallon-Baudry, & O’Regan, 2005). The superiority of performance when targets are organized in a “good Gestalt” underlines the classical role of chunking as a combinatory process of the attended objects in VSTM (Ripoll, Fiere, & Pelissier, 2005; Woodman et al., 2003).

Another important robust result is that the target dispersion does not reduce the capacity to memorize targets. Such a result underlines the capacity to allocate attention to different and non-contiguous locations. At first glance, this capacity seems to contradict the unitary conception of attention. However, it could be argued that certain properties of our material and procedure do not allow us to reject a unitary conception of attention. Furthermore, it is generally very difficult to distinguish a true division of attention across non-contiguous areas from a strategy in which a single attentional focus switches rapidly between several targets (Van Rullen, Carlson, & Cavanagh, 2007). In the following analysis of the dispersion effect, we therefore wanted to provide further arguments in favor of the competitive interaction model of attention, implying the possibility to allocate attention to different non-contiguous locations.

### Dispersion effects

The dispersion effect we obtained in a visual memory task involving genuine attentional processing constitutes a basic finding showing that (a) the processing of four targets is only weakly affected by the presence of spatially interleaving distractors; (b) the relative spatial location of targets and distractors is a strong determinant of the capacity to selectively encode and memorize targets in VSTM; and (c) the capacity to memorize the targets improves as the spatial dispersion increases.

One notable remark is that the dispersion effects can be explained neither by classical crowding and lateral masking nor by any other kind of sensory interactions between objects. Indeed, the dispersion effect was not observed in control groups 4 and 8. This dispersion effect is therefore a consequence of a genuine attentional effect which may be both feature-driven and goal-driven attention. As a whole, these results are consistent with the competitive interaction model (Caputo & Guerra, 1998; Desimone & Duncan, 1995; Mounts, 2000a, 2000b) since we observed that performance improved as distance between targets increased. In other words, when targets are in close spatial proximity, they draw largely or entirely on the same pool of processing structures. As a consequence, targets compete for their representation within the visual system and the capacity to memorize targets decreases as competition between them increases.

In the same line, the observed dispersion effect in control group 4with cueing of targets (Experiment 5) is a clear illustration of the efficiency of the spatial selective attention in our task. If the organism is precued about which locations to attend to, a saliency map may be configured before stimulus exposure. As proposed by Bundesen et al. (2005), neural structures (e.g., receptive fields at different levels of processing) contract around cued stimuli allowing parallel and independent processing of several cued objects. In the opposite case (without precue), the saliency map is not specifically configured and several (e.g., four) stimuli compete for representation by common neural structures. More generally, these results suggest that selectivity depends on both the spatial distance between targets and the attentional deployment that precedes targets onset.

The same dispersion effect was also observed based on feature-driven attentional allocation. In control group 8 (Experiment 4), the dispersion effect was clearly observed where target locations were not cued before target onset. In this condition, a distinctive feature property (color) discriminating targets from distractors has been used to allow for efficient selection and processing of the four targets. Such a finding underlines that the dispersion effect is not specific to only one kind of attentional deployment.

### Selection, processing, and dispersion effect

Many models and theories about visual attention do not clearly distinguish the capacity to allocate attention to several different locations (selection) from the capacity to encode and memorize the selected objects (consolidation). These two aspects of visual processing are combined in many visual tasks but it is not always easy to evaluate how each of them contributes to performance. For example, it is obviously more difficult to process two non-contiguous objects than to process one isolated object. However, any cost observed for the processing of two objects results from both the difficulty to deploy two attentional foci simultaneously and to process both objects simultaneously. The results we obtained suggest that targets’ dispersion may have two opposite effects linked to these two sequential stages. For example, the high level of performance obtained in condition C1 of the first experiment can be explained by an easy attentional allocation to a single area grouping the four targets compared to multiple non-contiguous locations. In this case, the decrease of targets’ dispersion seems to improve attentional allocation efficiency. When the difficulty to allocate attention and to select the targets is reduced by the introduction of color (Experiment 4), the process of consolidation in VSTM becomes the main source of variation. In this case, we observed that performance is very low in condition C1 (Experiment 4) and increases linearly as target dispersion increases. To sum up, it is easier to allocate attention to multiple foci when they are in close spatial proximity. On the other hand, the consolidation capacity for multiple objects should be more efficient as the distance between them increases.

The contribution of selection and consolidation could explain why such dispersion effect has been obtained whereas many previous results showed that participants encounter great difficulties in processing several targets among distractors, as for example, in the study of Palmer, Ames, and Lindsey (1993) which involved visual search of four targets interspersed with four distractors. At this point, it is essential to underline the importance of the task’s requirements and in particular the required perceptual level of processing. For low levels of processing (e.g., during a detection task), the selective component should play a more important role and performance should decrease as the dispersion of targets increases. On the contrary, for high levels of processing (memorization task), the second component (encoding and consolidation) should play a more important role. In this case, performance should increase as spatial distance between targets increases because competition between them is reduced. A related conclusion has been reached recently in independent research (Catena, Castillo, Fuentes, & Milliken, 2006; Vogel et al., 2005). Authors assumed that attention can only be split into discontinuous foci during high levels of processing (e.g., in a memory task). This theoretical conclusion is strongly supported by the empirical data we recently obtained. We tested again the dispersion effect with the same material as in the current experiments but we used a visual search task in which stimuli (four targets and four distractors) were presented for 100 ms and masked. As in the current experiments, targets were cued so that participants could restrict their search to only the target locations. In these conditions, the pattern of performance was the opposite of the pattern we obtained in the current study: Performance decreased as the level of dispersion increased. This clear dissociation suggests that attention can be deployed flexibly depending on the task. In the case of a memory intensive task, the visual system can memorize four targets among distractors efficiently even though such targets are located at non-contiguous locations.

Overall, our data are consistent with a multifocal attentional hypothesis as suggested by the attentional division hypothesis and biased competition model. Indeed, the findings reject the proposal of the unitary conception that the system is unable to process non-contiguoustargets simultaneously while filtering out embedded distractors (Posner et al., 1980). More important, we think that this multifocal capacity is set differently given the requirement of attentional allocation and processing.
